# *Gypsophila bermejoi* G. López: A possible case of speciation repressed by bioclimatic factors

**DOI:** 10.1371/journal.pone.0190536

**Published:** 2018-01-16

**Authors:** Miguel de Luis, Carmen Bartolomé, Óscar García Cardo, Julio Álvarez-Jiménez

**Affiliations:** 1 Departamento de Ciencias de la Vida, Facultad de Biología, C.Ambientales y Químicas Universidad de Alcalá, Alcalá de Henares (Madrid), Spain; 2 Empresa Pública de Gestión Ambiental de Castilla-La Mancha (GEACAM), Cuenca, Spain; Universita degli Studi di Napoli Federico II, ITALY

## Abstract

*Gypsophila bermejoi* G. López is an allopolyploid species derived from the parental *G*. *struthium* L. subsp. *struthium* and *G*. *tomentosa* L. All these plants are gypsophytes endemic to the Iberian Peninsula of particular ecological, evolutionary and biochemical interest. In this study, we present evidence of a possible repression on the process of *G*. *bermejoi* speciation by climatic factors. We modelled the ecological niches of the three taxa considered here using a maximum entropy approach and employing a series of bioclimatic variables. Subsequently, we projected these models onto the geographical space of the Iberian Peninsula in the present age and at two past ages: the Last Glacial Maximum and the mid-Holocene period. Furthermore, we compared these niches using the statistical method devised by Warren to calculate their degree of overlap. We also evaluated the evolution of the bioclimatic habitat suitability at those sites were the soil favors the growth of these species. Both the maximum entropy model and the degree of overlap indicated that the ecological behavior of the hybrid differs notably from that of the parental species. During the Last Glacial Maximum, the two parental species appear to take refuge in the western coastal strip of the Peninsula, a region in which there are virtually no sites where *G*. *bermejoi* could potentially be found. However, in the mid-Holocene period the suitability of *G*. *bermejoi* to sites with favorable soils shifts from almost null to a strong adaptation, a clear change in this tendency. These results suggest that the ecological niches of hybrid allopolyploids can be considerably different to those of their parental species, which may have evolutionary and ecologically relevant consequences. The data obtained indicate that certain bioclimatic variables may possibly repress the processes by which new species are formed. The difference in the ecological niche of *G*. *bermejoi* with respect to its parental species prevented it from prospering during the Last Glacial Maximum. However, the climatic change in the mid-Holocene period released this block and as such, it permitted the new species to establish itself. Accordingly, we favor a recent origin of the current populations of *G*. *bermejoi*.

## Introduction

Polyploidy is a spontaneous evolutionary force responsible for the appearance of new species and it is a key process in the diversification of species, although it is more widespread in plants than in animals [1,2]. Polyploidy is characterized by the presence of two or more sets of paired or homologous chromosomes per nucleus. Allopolyploids are generally of greater interest than autopolyploids since they generate more genetic diversity and therefore, greater hybrid vigor. Indeed, in both natural and synthetic allopolyploids one parental genome is preferentially expressed over the other. New phenotypes emerge in allopolyploid species due to genetic and epigenetic phenomena, and they generally occupy environments that differ from those occupied by their progenitors.

Speciation by hybridization may be considered as macro-mutation, capable of displacing the resulting hybrid to regions quite distant in the hyperspace of the possible phenotypes. The intermediate phenotypes of these hybrids may be distinct from the optimal phenotype of the parental species. Moreover, in addition to the advantage of heterozygosity, the polyploid hybrids that form may experiment the benefits of reproductive isolation and the possibility of expressing extreme traits [3–28]. Thus, it makes sense to think that this will affect the ecological niche of the hybrid species.

Species Distribution Models allow the ecological preferences of a species within a geographic space to be visualized and they have been used to study a wide range of ecological issues. For instance, such models have been successfully employed to study the influence of bioclimatic variables on the distribution of plant pathogens, their biological invasion and the influence of human activity on them. They have also been used to evaluate the effects of climate fluctuations on ecosystems, or the effectiveness of conservation plans for certain plant species [29–43]. We believe that these tools can also shed some light on the evolution of species and in particular, on the possible effects of climatic change on the process of sympatric speciation that gave rise to *G*. *bermejoi*.

Accordingly, here we have analyzed a tetraploid allopolyploid (*Gypsophila bermejoi* G. López) [44] from an ecological viewpoint, adopting the perspective of its ecological niche (according to the definition of Hutchinson [45,46]) to assess the influence of abiotic (climatic) factors on the evolution of this particular taxon (*G*. *bermejoi*). We aimed to demonstrate that the ecological niche for the allopolyploid species is markedly different from that of the parental species and that this has had important evolutionary consequences. We think that bioclimatic factors could prevent speciation from occurring effectively in some cases. Conversely, climatic change could reverse such situations, facilitating the appearance of a new species.

## Materials and methods

### Study area and species

**Fig 1:**
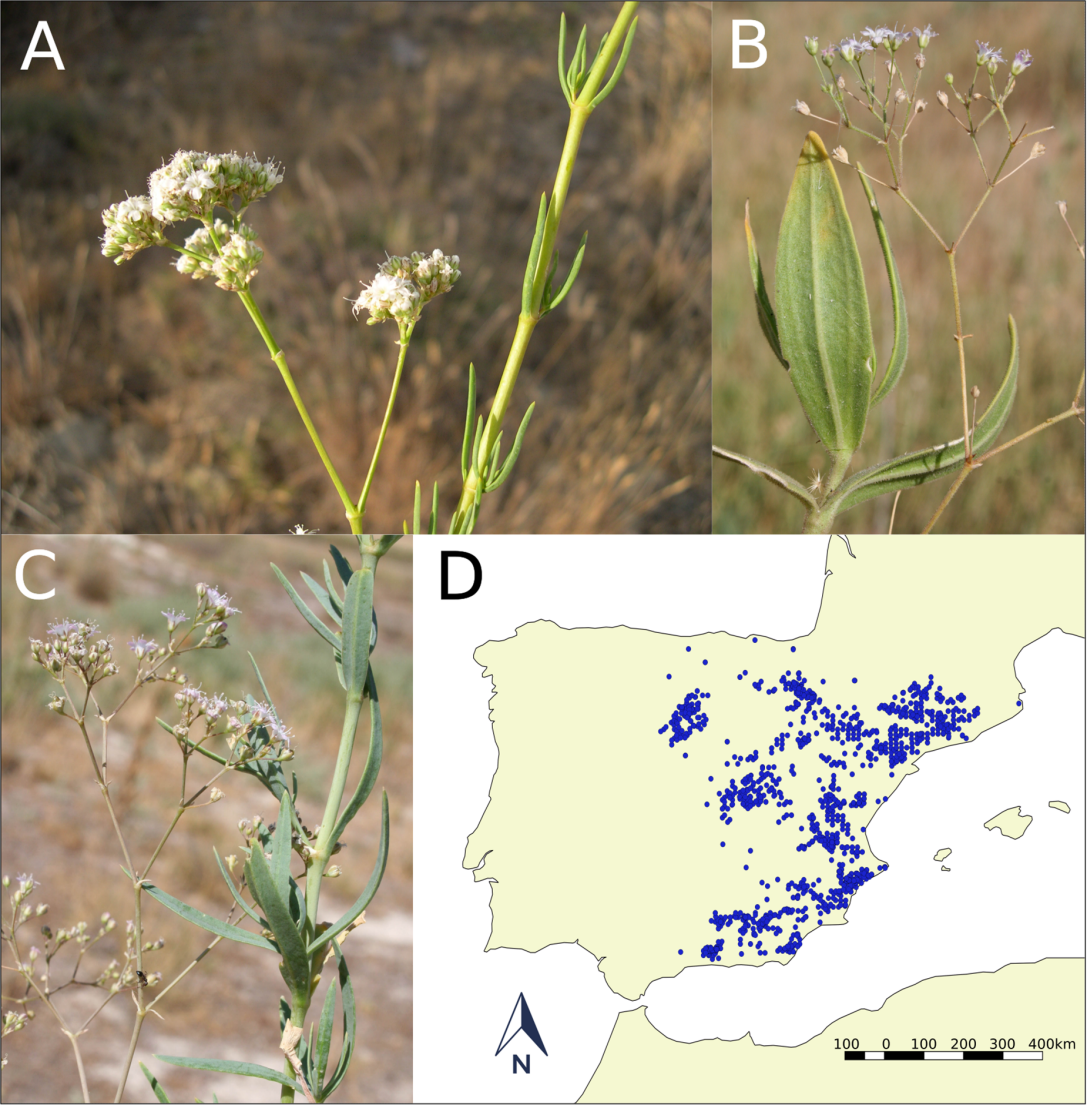
Appearance of *G*. *struthium* subsp. *struthium* (A), *G*. *tomentosa*(B) and *G*. *bermejoi* (C). Map of the Iberian Peninsula showing the presence of *Ononis tridentata* L. (D), a plant that is indicative of gypsum and gypsiferous loam soils.

The area studied here is the Iberian Peninsula, a territory located between latitudes 36°00’08"N—43°47’38”N and longitudes 9°29’00"O—3°19’00”E, with an approximate extension of 623,000 km^2^. As indicated, we centered on the plants endemic to “the gypsum steppes” in the Iberian Peninsula. These gypsum outcrops are concentrated in the eastern half of the territory and they are under the influence of a Mediterranean climate (see map Fig 1D).

In this study, we focus on the speciation of *G*. *bermejoi*, an allopolyploid species derived from *G*. *struthium* subsp. *struthium* and *G*. *tomentosa* [44], three taxa of the genus *Gypsophila* (*Caryophyllaceae)*. *Gypsophila bermejoi* G. López (2n = 68) is an allopolyploid endemic species of the Iberian Peninsula that is found on verges and slopes in areas of gypsum soil. The parental species are *G*. *struthium* subsp. *struthium* L. (2n = 34) and *G*. *tomentosa* L. (2n = 34) [44], and while *G*. *bermejoi* is distributed in areas where the former may be found, it occupies niches with different ecological characteristics. All these perennial plants are endemic to the Iberian Peninsula, inhabiting salt-rich substrates and fundamentally, gypsum soils (Fig 1A to 1C).

All gypsophytes must face difficult environmental conditions, the most notable of which are the strong xericity of their habitat, the existence of a crusty surface and important nutritional imbalances typical of these saline soils [47]. They are very interesting biochemically, not least for their tendency to accumulate certain minerals and for their capacity to synthesize secondary metabolites of interest. As such, the production of antioxidants like phenols and flavonoids by *G*. *struthium* [48] and that of saponins by *G*. *bermejoi* have been studied [49,50]. The particular ecological conditions faced by gypsophytes make them very highly adapted to their environment and hence, the abundance of endemic species. Some of these endemisms are very local and of recent origin (microendemisms/neoendemisms). The strong influence of abiotic factors and the limited areas they occupy mean these plants are considered an interesting model to study plant evolution and speciation [48].

### Data sources

The modelling of ecological niches using MaxEnt requires two types of data to be entered, data regarding the presence of the species and a series of environmental variables. In this study, we used data regarding the presence of the species based on the georeferenced registers of the Global Biodiversity Information Facility (GBIF) database. This infrastructure provides access to information about living beings, containing data on more than 1.6 million species [51].

The WorldClim portal (www.worldclim.org) provides data on 19 bioclimatic variables widely considered to be significant from a biological point of view when modelling areas of distribution [52]. The data appears in the form of world maps for the different bioclimatic variables and they are the result of extrapolating values taken from tens of thousands of meteorological stations distributed around the world. Maps of these bioclimatic variables for the past and future can also be found at this platform, generated using different climate models. In this work we used the bioclimatic maps for the mid-Holocene and the Last Glacial Maximum, generating predictions with the Community Climate System Model (CCSM4). This model simulates the global climate by unifying four separate models of the atmosphere, land, oceans and sea-ice [53].

In this study, the potential distribution of *G*. *bermejoi* and its parental species was initially obtained based on the bioclimatic variables. In order to take into account factors related to the soil, we studied the suitability of the habitat in those locations with soils that favor these species and their evolution since the Last Glacial Maximum. In order to define these sites, we used data on the presence of *Ononis tridentata* L., a common and widespread species that lives on more or less pure Gypsum soils or on gypsiferous loams [54]. We also obtained this data from the GBIF.

### Maximum entropy (MaxEnt) models

The application of the principal of Maximum Entropy (MaxEnt) to the modelling of ecological niches and areas of potential distribution follows the work of Phillips [55] and subsequent modifications [56]. The required algorithms were implemented by employing a graphic interface that aids their use (freely available at http://www.cs.princeton.edu/~schapire/maxent/) and the most recent version of the program available was used (MaxEnt 3.3.3: for a detailed explication of the method see [55–59]). Essentially, this tool estimates a probability distribution for the presence of the species in function of the environmental variables selected. The data is entered as a data file of the presence of the species and a series of maps of the environmental variables considered to be significant for that particular species. Given the starting data, the program generates a map in which each pixel is assigned a value between 0 and 1. These values can be interpreted as an index of suitability of the habitat for that particular species [55], an interpretation that we adopt in this study.

We dealt with the autocorrelation issues by eliminating redundant presence in each pixel on the scale of the bioclimatic variables used. We used 70% of the presence records to train the models and 30% to test them. To minimize the number of variables we used only bioclimatic variables to generate the models. Given the strict gypsophillic nature of these plants, they can only be present in gypsum soils. We generated simple models and then incorporated them into a single layer with the information regarding the suitable soils. Thus, we will only have values of the bioclimatic suitability in the places where these plants may be present. This procedure is detailed in section 2.6 (see below).

The MaxEnt software also allows response curves to be generated for the species relative to each of the variables used to establish the model. These are graphs in which the suitability of the species to the habitat is represented in light of a given variable. The response curves are useful tools to evaluate the optimal values, the tolerance intervals and the thresholds of the different variables for a given species. MaxEnt offers two types of response curves, the first are the response curves obtained when using all the variables employed in the model together. The second are the response curves generated when the model focuses only on the individual variables. In this study we analyzed the former type of response curves.

### Selection of the variables and the “feature” functions

When elaborating models using the MaxEnt method the correlation between the predictors should be minimized [58]. From a series of biologically plausible predictors, we eliminated some that are strongly correlated, a process we performed using a clustering algorithm. A dendrogram was obtained that groups the variables in function of their degree of correlation according to the Pearson coefficient, r. Furthermore, we ran the models with all the variables to perform a jack-knife test of the contribution of the variables to the model. After combining the information obtained in both processes, we selected four bioclimatic variables for our models (Table 1).

**Table 1:** List of the environmental variables selected to implement the SDM.

Variable	Source	Resolution
bio4—the dispersion of seasonal temperatures	WorldClim	2.5’
bio6 –the minimum temperature in the coldest month	WorldClim	2.5’
bio13 –the rainfall in the wettest month	WorldClim	2.5’
bio14 –the rainfall in the driest month	WorldClim	2.5’

In choosing the functions “feature”, we deactivated the functions hinge and threshold. In this way, the response curves obtained were easier to interpret and they were better adjusted to the Ecological Theory of the niches [43].

### Model evaluations and comparison of the ecological niches using Warren statistics

We evaluated the models using two validation methods: the Area Under the Curve (AUC) [60] and the True Skill Statistics (TSS) [61].

As well as a direct interpretation of the maps generated by the models, the differences estimated between the niches was quantified using the overlap of niche “I” and “D”, as proposed by Warren [62]. This statistical analysis was carried out in the “R” programming language [63] of the Phylocom package, entering the data via the maps generated by the MaxEnt models (as an ASCII file) and recovering the data as elements in a matrix that compare the three *Gypsophila* species. As such, we obtained a 3 x 3 matrix the diagonal of which is void as it would contain the comparison of each species with itself. The values above the diagonal correspond to those based on Schoeners’ “D” [64], while those below the diagonal refer to the Hellinger’s distances [65]. As seen below, both these indices show larger differences for *G*. *bermejoi* relative to the parental species than between the parental species themselves.

### Study of suitability at sites with gypsum soils

The maps we generated using MaxEnt indicate the suitability of the bioclimatic habitats in the study area. However, true suitability would be zero in most of the territory, given the absence of adequate soil conditions. To define suitability in the gypsiferous areas we need to know their locations. Outcrops of this type do not usually cover large extensions and at the scale of the variables used, most of these outcrops would appear as mere points. Therefore, to trace the areas with gypsum soils we chose to use records of the presence of a gypsum indicator plant, *Ononis tridentata L*. [66–68]. This species is adequate for this purpose as like the species under study, it can grow on gypsum soils as well as on gypsiferous loams. We used QuantumGIS [69] to represent the georeferenced presence of *O*. *tridentata* L. and the pattern obtained coincided perfectly with the diverse maps published of Iberian gypsum soils [67].

Using the same GIS, QuantumGIS, we obtained the suitability values from the models for those gypsum sites for each species today, in the mid-Holocene period and at the Last Glacial Maximum. Subsequently, we represented these values through box-plots, which allowed us to compare the suitability of the three species at any given time. It is important to note that the atypical values (outliers) could be indicative of sites with gypsum soils that are bioclimatically suitable, although the predominant conditions of the region as a whole are unfavorable.

## Results

Results were obtained from the models generated with MaxEnt for the three **studied** species: *G*. *bermejoi*, *G*. *tomentosa* and *G*. *struthium* subsp. *struthium*. The AUC values obtained were 0.965(*G*. *bermejoi*), 0.911 (*G*. *tomentosa*) and 0.907(*G*. *struthium struthium*), all of which were sufficiently high to accept the models generated. For the same species, the TSS values were 0.810 (*G*. *bermejoi*), 0.668 (*G*. *tomentosa*) and 0.584 (*G*. *struthium struthium*), with values above 0.6 considered to be good and 0.2–0.6 to be fair to moderate.

### Potential distribution of the *Gypsophila* species under study

**Fig 2:**
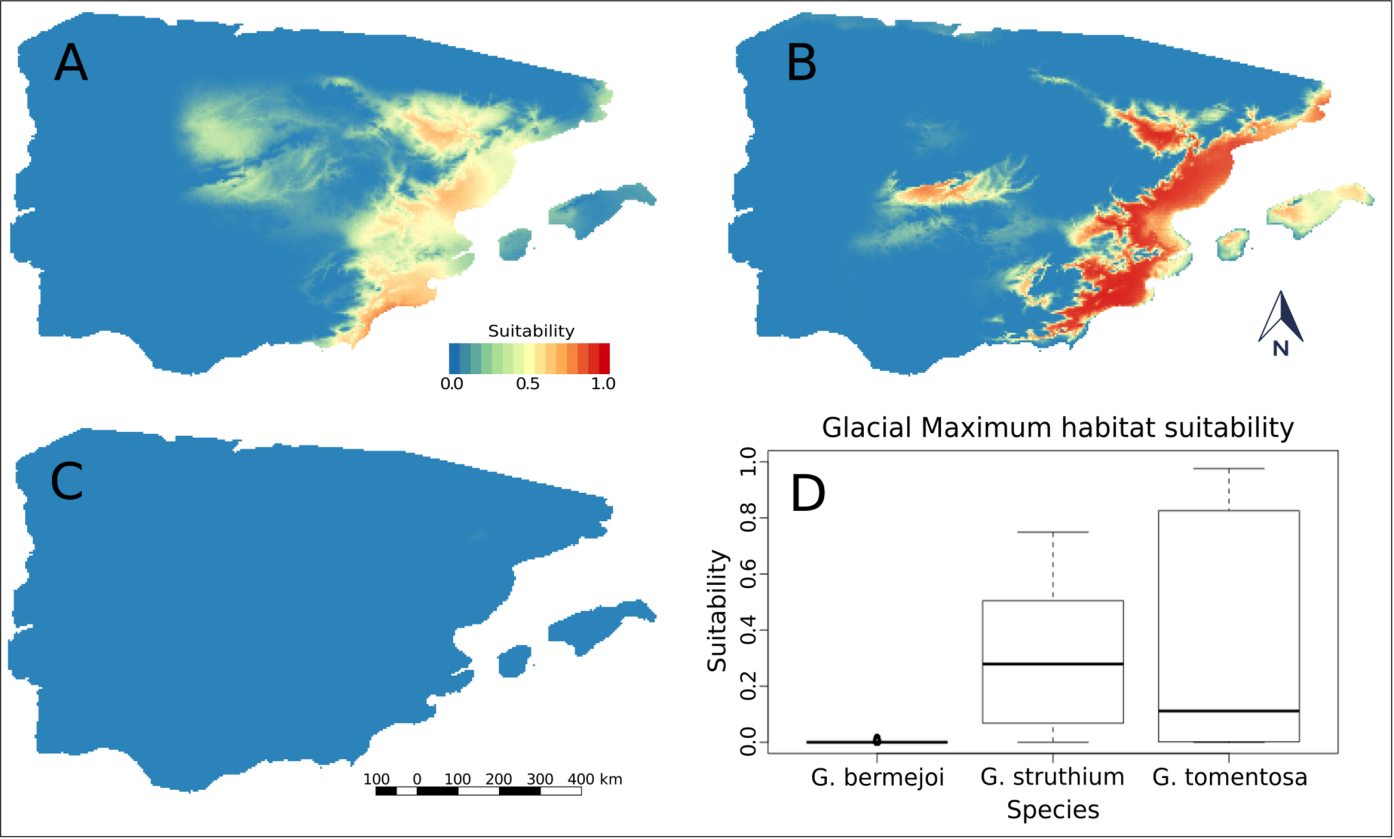
Habitat suitability predicted for *G*. *tomentosa* (A), *G*. *struthium* subsp. *struthium* (B) and *G*. *bermejo*i (C) during the Last Glacial Maximum. These are the potential areas according to the bioclimatic variables for the three taxa. All these plants are strict gypsophytes and the real values of suitability are 0 in the locations without suitable soils. (D) shows the differences in bioclimatic suitability for the three species. To produce these box plots, only those values from locations with favorable soils (see map Fig 1D) have been taken into account. Notice that both, maps and box plots, show a suitability of almost 0 for *G*. *bermejoi* during this period.

When we projected these models onto the climatic conditions calculated for the Last Glacial Maximum, we obtained similar potential distributions for *G*. *tomentosa* and *G*. *struthium* subsp. *struthium*. In both cases, the species seemed to take shelter on the eastern and south eastern coastal strip of the Iberian Peninsula, this zone extending into inland areas along the Ebro Valley (Fig 2A and 2B). There was also an area of somewhat higher suitability for both these species south of the Central System. The most notable difference between the potential distributions of these two species was an area in the northwest region of the Central System where there was some degree of suitability for *G*. *struthium* subsp. *struthium* (Fig 2A). There were virtually no areas potentially suitable for *G*. *bermejoi* in the same period as the model yielded a signal that approximated to zero (Fig 2C).

**Fig 3:**
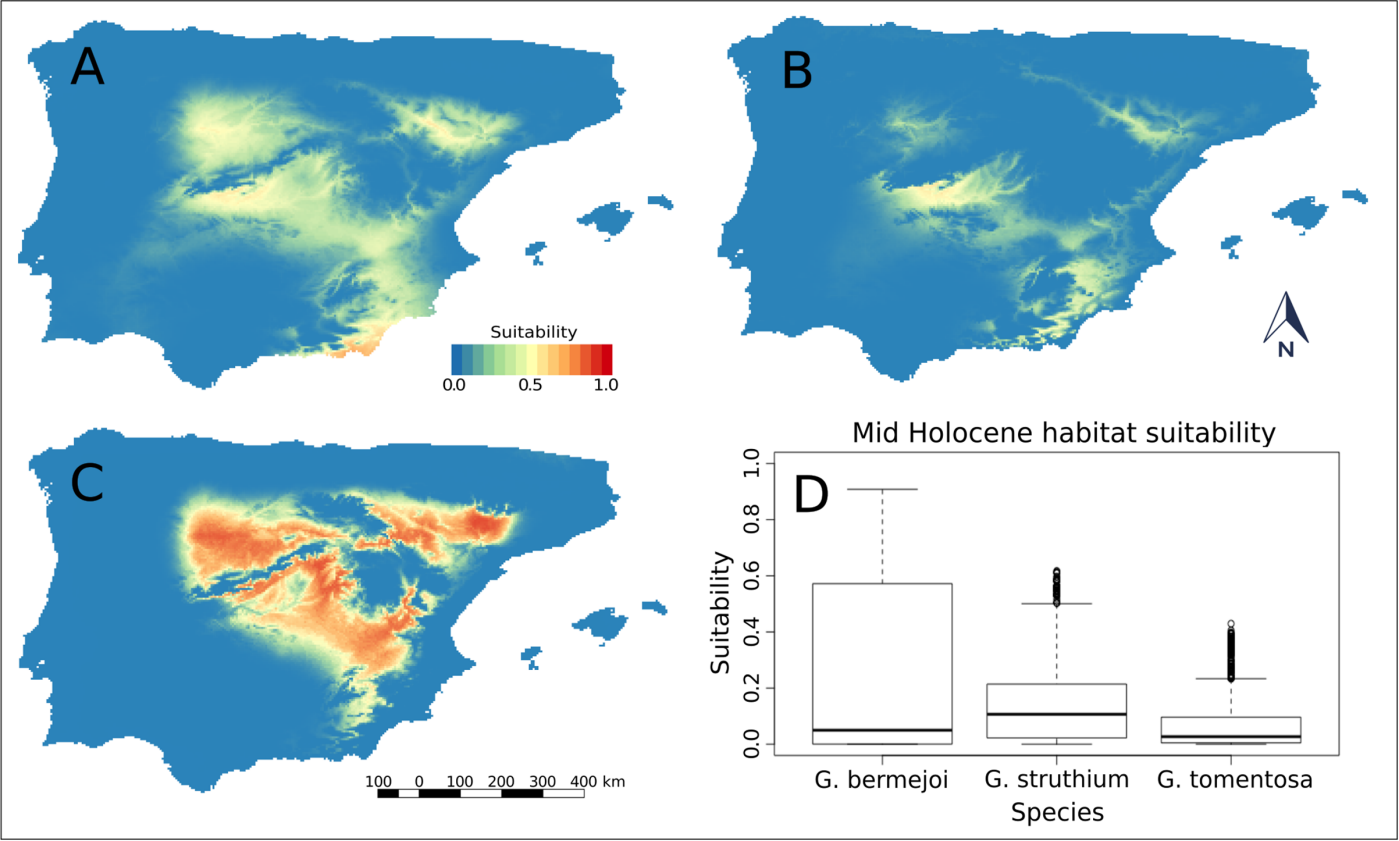
Habitat suitability predicted for *G*. *tomentosa* (A), *G*. *struthium* subsp. *struthium* (B) and *G*. *bermejo*i (C) during the Mid Holocene. In this case, (D) shows the variation of the habitat suitability for the three taxa with the climatic change from Last Glacial Maximum to Mid Holocene. For *G*. *struthium* subsp. *struthium* and *G*. t*omentosa*, the medians have decreased. However, there are some outliers indicating locations with a relative high bioclimatic suitability. For *G*. *bermejoi*, those values have increased dramatically.

The situation changed markedly for the three species in the mid-Holocene period, with the greatest contrast for *G*. *bermejoi*. The potential distribution of *G*. *tomentosa* narrowed in the northernmost part of the eastern coastal strip, while it was maintained in the Tagus valley and a more or less favorable area appeared in the North Sub-Plateau (Fig 3B). The potential distribution of *G*. *struthium* subsp. *struthium* (Fig 3A) changed significantly, its habitat suitability intensifying and expanding in both the North and South Sub-Plateau. There was also an isolated area suitable for this species in the Ebro Valley, while its potential distribution in the eastern coastal strip became restricted to the southernmost part, communicating with the South Sub-Plateau area (Fig 3B). Finally, an important area of strong *G*. *bermejoi* suitability appeared in the Ebro Valley, and another emerged in the Duero Valley and in the central and south-eastern part of the Peninsula. There were no major disjunctions between these three zones (Fig 3C).

**Fig 4:**
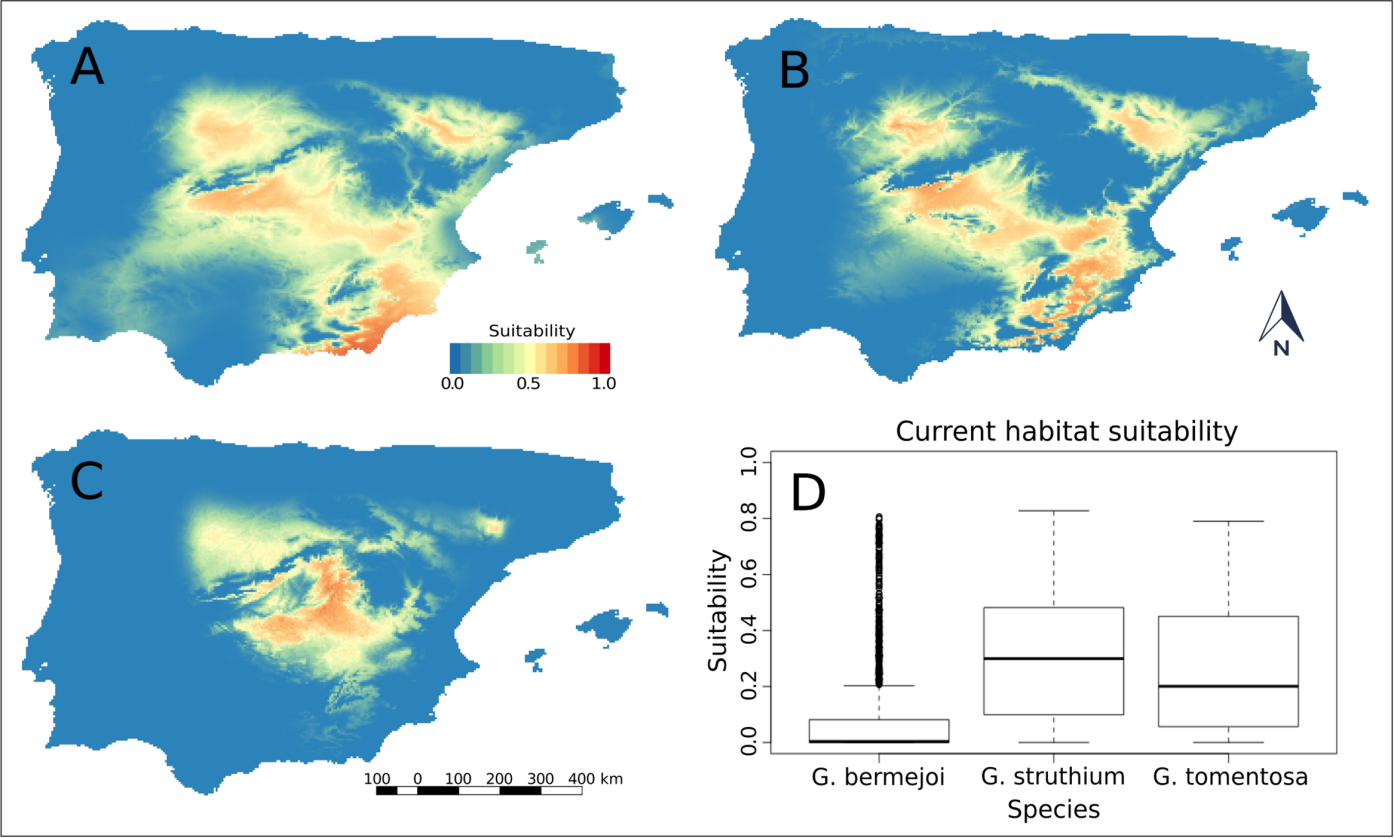
Habitat suitability predicted for *G*. *struthium* subsp. *struthium* (A), *G*. *tomentosa* (B) and *G*. *bermejo*i (C) using current climatic data. For *G*. *bermejoi* (D) shows the emergence of several sites with atypically high values, a behavior consistent with its highly endemic character.

In current times, *G*. *tomentosa* was seen to have a more extensive area of suitability relative to the mid-Holocene period, with a significant increase in the predicted values of habitat suitability. *G*. *struthium* subsp. *struthium* also showed a similar behavior in the transition from the mid-Holocene period to the present. By contrast, the potential distribution *G*. *bermejoi* was more restricted and it was reduced considerably in the Ebro Valley (Fig 4A, 4B and 4C). The predicted values of habitat suitability for all the zones indicated in the map were generally lower. We should stress that most previous models were developed using only bioclimatic predictors. Bioclimatic suitability is a necessary but not sufficient condition to determine the potential distribution of these species in a given area and at a given time.

### Comparison of ecological niches

We compared the niches of the three species using the Warren statistic, which yielded results consistent with the information obtained from the models generated with MaxEnt. In fact, the index of ecological niche similarity was high for the two parental species, explaining the overlap of their potential areas of distribution. However, significantly lower values were obtained when the niches of these species were compared to that of *G*. *bermejoi* (Table 2). In other words, the ecological behavior of *G*. *bermejoi* differed substantially from that of its parental species.

**Table 2:** Test statistics D and I of the niche overlap proposed by Warren and based on Schoener’s D and Hellinger’s distances. The niche overlap is high when comparing the parental species. But it decreases when comparing one of the parental species with the allopolyplid species (*G*. *bermejoi*).

Taxa	D	I
*G*. *tomentosa—G*. *struthium struthium*	0.7311903	0.9072852
*G*. *tomentosa—G*. *bermejoi*	0.4396845	0.6772885
*G*. *struthium struthium—G*. *bermejoi*	0.4957315	0.7369228

### Suitability at sites with favorable soils

*O*. *tridentata* is a common plant indicative of gypsum and gypsiferous loam soils, and it is found widely on these types of substrates. These soils are also those that favor the presence of the three species of Gypsophila under study. Therefore, the areas where *O*. *tridentata* is present on a map would also indicate sites with soils favorable for the presence of *G*. *tomentosa*, *G*. *struthium* subsp. *struthium* and *G*. *bermejoi* (Fig 1D).

During the Last Glacial Maximum, the values of bioclimatic suitability at the sites with gypsiferous soils were extremely low for *G*. *bermejo*i (Fig 2D) and in addition to the low values obtained, the weak dispersion of such values was noteworthy. By contrast, we found that these favorable soils had significantly superior values of bioclimatic suitability for *G*. *struthium* subsp. *struthium* and *G*. *tomentosa*.

A summary of the statistics for these species at these sites in the Last Glacial Maximum are shown in Table 3, where the large differences in the median, mean and maximum values between the allopolyploid species and its parental species are noteworthy.

**Table 3:** Evolution of the habitat suitability values (means, medians and standard deviations) for the three *Gypsophila* taxa during the climatic periods included in this research. The values were taken in the locations where *O*. *tridentata* is present, to make sure that soils are favorable for the presence of gypsophytes. Notice the variation in several orders of magnitude in the transition from the Last Glacial Maximum to the Mid Holocene for *G*. *bermejoi*.

	Glacial Maximum	Mid Holocene	Current Climate
**Taxon**	***Mean***	***Mean***	**Mean**
*G*. *bermejoi*	0.0005139	0.26596	0.09135
*G*. *struthium struthium*	0.28835	0.14009	0.29627
*G*. *tomentosa*	0.33960	0.061178	0.25907
	***Median***	***Median***	***Median***
*G*. *bermejoi*	0.0000400	0.04989	0.00345
*G*. *struthium struthium*	0.27889	0.10667	0.29959
*G*. *tomentosa*	0.11135	0.026880	0.20068
	***SD***	***SD***	***SD***
*G*. *bermejoi*	0.00119729	0.3253359	0.1700603
*G*. *struthium struthium*	0.2201172	0.1380697	0.2185862
*G*. *tomentosa*	0.3924741	0.08020903	0.2191469

We used box-and-whisker plots to represent the suitability of sites with soils appropriate for the development of the three species under study during the mid-Holocene period. The habitat suitability values for *G*. *bermejoi* displayed greater dispersion (Fig 3D), with an overall significant increase in all values and a much more marked maximum value. By contrast, the values of suitability decreased considerably for *G*. *struthium* subsp. *struthium* and *G*. *tomentosa*, with the latter species showing a sharper decrease. Nevertheless, there were sites at which relatively high suitability for both species persisted (shown on the plot as outliers), a behavior that might be expected for highly endemic species. The values of bioclimatic suitability for *G*. *bermejoi* were extremely low at these sites, in addition to showing a notably lower dispersion. Alternatively, we found that the bioclimatic suitability for *G*. *struthium* subsp. *struthium* and *G*. *tomentosa* in these favorable soils was significantly higher.

We would like to highlight the strong difference between the suitability values at these sites for the three species during the mid-Holocene and the Last Glacial Maximum, as shown in the statistical summary for this period (Table 3). Suitability was markedly enhanced for *G*. *bermejoi*, with the median and mean increasing by several orders of magnitude. By contrast, the suitability values decreased for the other two species, although there were still several sites with gypsum or gypsiferous loam soils at which relatively high suitability values persisted.

In the current climatic conditions, the suitability values for *G*. *bermejoi* clearly decreased further (Fig 4D), although they were still significantly higher than during the Last Glacial Maximum. The emergence of several sites with atypically high values was striking, a behavior consistent with its highly endemic character. Conversely, there was a tendency for *G*. *struthium* subsp. *struthium* and *G*. *tomentosa* to coincide, in the absence of *G*. *bermejoi*, and the suitability general increased for both species. There were many points with suitability values equivalent to those atypically high values during the mid-Holocene for both species. Again, a more detailed summary of the data mentioned above is provided (Table 3).

### Species response curves

The response curves generated by MaxEnt are useful to evaluate the ecological behavior of different taxa. One of the goals of our work was to study the behavior of an allopolyploid species with respect to its parental species and in this sense, the variables that showed the greatest differences were bio 4 and bio 6. In terms of bio 4, the tendency of *G*. *bermejoi* towards this variable were not merely different but they were completely opposite to both *G*. *tomentosa* and *G*. *struthium struthium*. By contrast, bio 6 showed that *G*. *bermejoi* had low tolerance to the annual drought period (See supplementary material), which was probably a determining factor in the almost null bioclimatic suitability of this species in the last Maximum Glacial (Fig 2C and 2D).

## Discussion

The results presented here reflect a panorama during the Last Glacial Maximum in which the parental species (*G*. *tomentosa* and *G*. *struthium* subsp. *struthium*) appear to be capable of crossing, yet *G*. *bermejoi* remains absent, as reflected by the distinct types of evidence. In the first place, *G*. *tomentosa* and *G*. *struthium* subsp. *struthium* may be found in similar areas of the maps and in both cases, there is a strip in the proximal western coastal region of the Peninsula that apparently served as a glacial refuge. In this area there is apparently no or very little *G*. *bermejoi*, for which there are very low suitability values and only in a narrow strip. Comparing the ecological niches using the “I” and “D” parameters proposed by Warren [62] suggests a stronger overlap between *G*. *tomentosa* and *G*. *struthium* subsp. *struthium* than with *G*. *bermejoi*. This strengthens the possibility of sympatry between the parental species and a possible incompatibility of *G*. *bermejoi* with its parental species under certain bioclimatic conditions. The response curves obtained also highlight a distinct ecological behavior of the parental species to that of *G*. *bermejoi*.

Alternatively, we know that *G*. *tomentosa* and a *G*. *struthium* subsp. *struthium* crossed recently to produce the hybrid *G*. *x castellana* Pau [1,16]. Unlike *G*. *bermejoi*, this hybrid is relatively sterile, although its existence demonstrates that both species share insect pollinizers. It would not be unreasonable to think that if the bioclimatic conditions permit (both species might have coincided at certain sites during the last Glaciation), they would also permit their pollinators to be present. The experimental studies available regarding pollination refer to *G*. *struthium*, indicating that the groups that participate most actively in this process are the Coleoptera of the *Mordellidae* family and the Diptera of the *Bombyliidae* family, with a mean number of insect visits per plant of 70.90 and 17.43 each 10 minutes, respectively [70]. The pollinating insects were determined at the family level, which makes it difficult to generate models of the niches and of the potential distribution of the pollinators. Nevertheless, it is known that both are families with species that tolerate a wide range of bioclimatic conditions [71].

With respect to the germination of these species, it is known that they follow an opportunistic behavior, with dormant periods and where the availability of water is the main limiting factor [72]. Our response curves appear to point to rainfall in the driest month as a possible limiting factor for germination.

Given these considerations, it would appear reasonable to accept the absence of *G*. *bermejoi* in the Peninsula during the Last Glacial Maximum and the co-existence at various sites of *G*. *tomentosa* and *G*. *struthium* subsp. *struthium*. We believe it is possible that these two species crossed during this period and produced seeds of an allopolyploid plant similar to *G*. *bermejoi*. Nevertheless, the climatic conditions in the Last Glacial Maximum would not have permitted this plant to become established given the marked difference in the niche with respect to that of *G*. *tomentosa* and *G*. *struthium* subsp. *struthium*. As such, certain bioclimatic factors could have acted as a brake on the process of speciation.

Various studies indicate an important climatic change was associated with the end of the glacial period, which in general terms provoked a warmer and more humid climate during the mid-Holocene period [73]. The models of the potential distributions that we obtained also show notable changes in the species studied with respect to the Last Glacial Maximum. The most notable difference was the apparent irruption of *G*. *bermejoi* over vast areas of the Iberian Peninsula. It should be remembered that these plants would be restricted to sites with gypsiferous soils. On estimating the climatic suitability of these sites we detect a notable increase in these values, whereas the suitability of *G*. *tomentosa* and *G*. *struthium* subsp. *struthium* at such sites generally decreases. Yet it is important to indicate the existence of atypically high suitability values at a given number of sites for both species, behavior expected for endemic species. We believe that by varying the influence of climatic factors that night be repressing the establishment of *G*. *bermejoi* during the Last Glacial Maximum, the seeds of this allopolyploid hybrid could develop and complete their life cycle. As such, we favor a recent origin for the populations of this species in the Iberian Peninsula.

In our current climatic conditions, this tendency appears to have been inverted and the climatic suitability of *G*. *bermejoi* at sites with gypsiferous soils is weaker, whereas it is on the rise for *G*. *tomentosa* and *G*. *struthium* subsp. *struthium*. Obviously, the extremes of the Last Glacial Maximum have not returned and the suitability values are atypically high for *G*. *bermejoi* at several sites, indicative of its endemic nature. The climatic suitability of *G*. *tomentosa* and *G*. *struthium* subsp. *struthium* is also enhanced in these soils, which can in part explain their higher abundance with respect to *G*. *bermejoi*.

## Conclusions

From the results obtained we can draw the following conclusions:

a) Although they occupy similar areas, there are differences in the ecological niches of the parental species and the hybrid allopolyploid. While it is logical that the ecological niches of the parental species are similar, enabling genetic exchange between them, there must also be some degree of sympatry. As such, the ecological niche of the hybrid should also maintain some features of that of its parental species. Nevertheless, the results of the MaxEnt models and of the Warren statistics appear to indicate otherwise. These differences in the ecological niches are notable and they may be relevant from an evolutionary point of view. In the light of the studies into the genetics and epigenetics of the hybrid species, we believe this not to be an isolated phenomenon. Indeed, there is considerable evidence of the appearance of extreme phenotypes in allopolyploids, both when these hybrids arise naturally or when they are produced artificially [74].

b) The second conclusion we can draw from these studies is related to the possible evolutionary consequences of these differences. It appears reasonable that a bioclimatic factor or factors could have repressed the speciation of *G*. *bermejoi*. It appears plausible that we are dealing with a case of post-glacial speciation and thus, a relatively recent event. As such, the climate change in the transition from the Last Glacial Maximum to the mid-Holocene permitted the hybrid to survive, even though the conditions during glaciation did not favor this allopolyploid species. Given that speciation by allopolyploidy is relatively frequent in plants, we believe it would be interesting to carry out further studies to determine whether this is a more extended phenomenon in other species.

## Supporting information

S1 Fig Response curves of *G*. *bermejoi* to the seasonal temperature dispersion (bio4, expressed as standard deviation *100), the minimum temperature of the coldest month (bio6, expressed in °C * 10), the rainfall in the wettest month (bio13, expressed in mm) and the rainfall in the driest month (bio14, also in mm). (TIF)

S2 Fig Response curves of *G*. *struthium* subsp. *struthium* to the seasonal temperature dispersion (bio4, expressed as standard deviation *100), the minimum temperature in the coldest month (bio6, expressed in °C * 10), the rainfall in the wettest month (bio13, expressed in mm) and the rainfall in the driest month (bio14, also in mm). (TIF)

S3 Fig Response curves of *G*. *tomentosa* to the seasonal temperature dispersion (bio4, expressed as standard deviation *100), the minimum temperature in the coldest month (bio6, expressed in °C * 10), the rainfall in the wettest month (bio13, expressed in mm) and the rainfall in the driest month (bio14, also in mm). (TIF)

S1 File MaxEnt output (suitability) for gysum soils as indicated by the presence points of *Ononis tridentata* for *Gypsophila bermejoi*, *G*. *struthium struthium* and *G*. *tomentosa* in the Last Glacial Maximum. (CSV)

S2 File As S4, in the Mid Holocene. (CSV)

S3 File As S4, in the current climate. (CSV)
