# Supplementary figures and images for: Gypsophila bermejoi G. López: A possible case of speciation repressed by bioclimatic factors
Source: PLoS One. 2018 Jan 16;13(1):e0190536. doi: 10.1371/journal.pone.0190536 (PMC5770026; doi:10.1371/journal.pone.0190536)

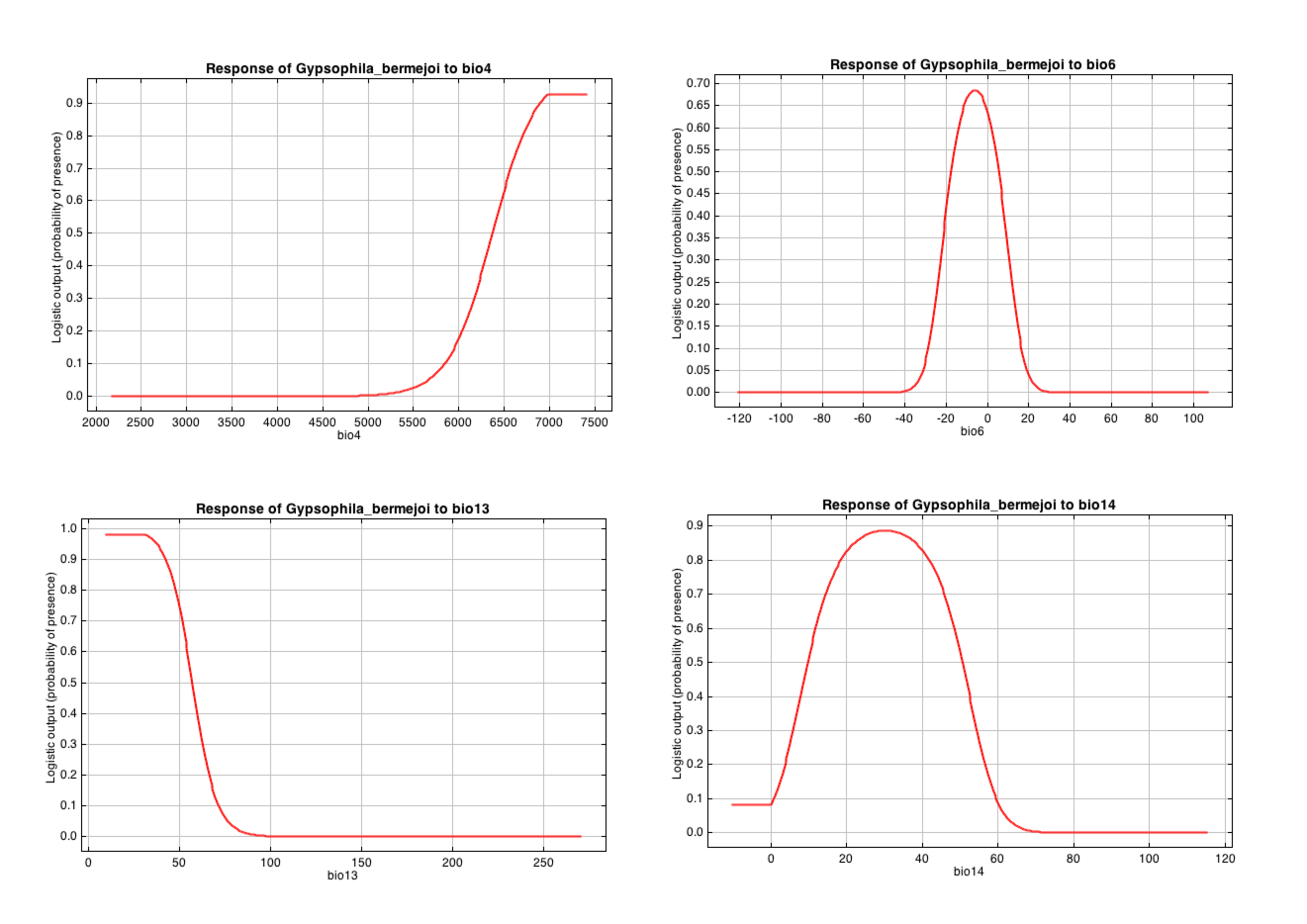

Supplement: S1 Fig — (TIF) [file pone.0190536.s001.tif]

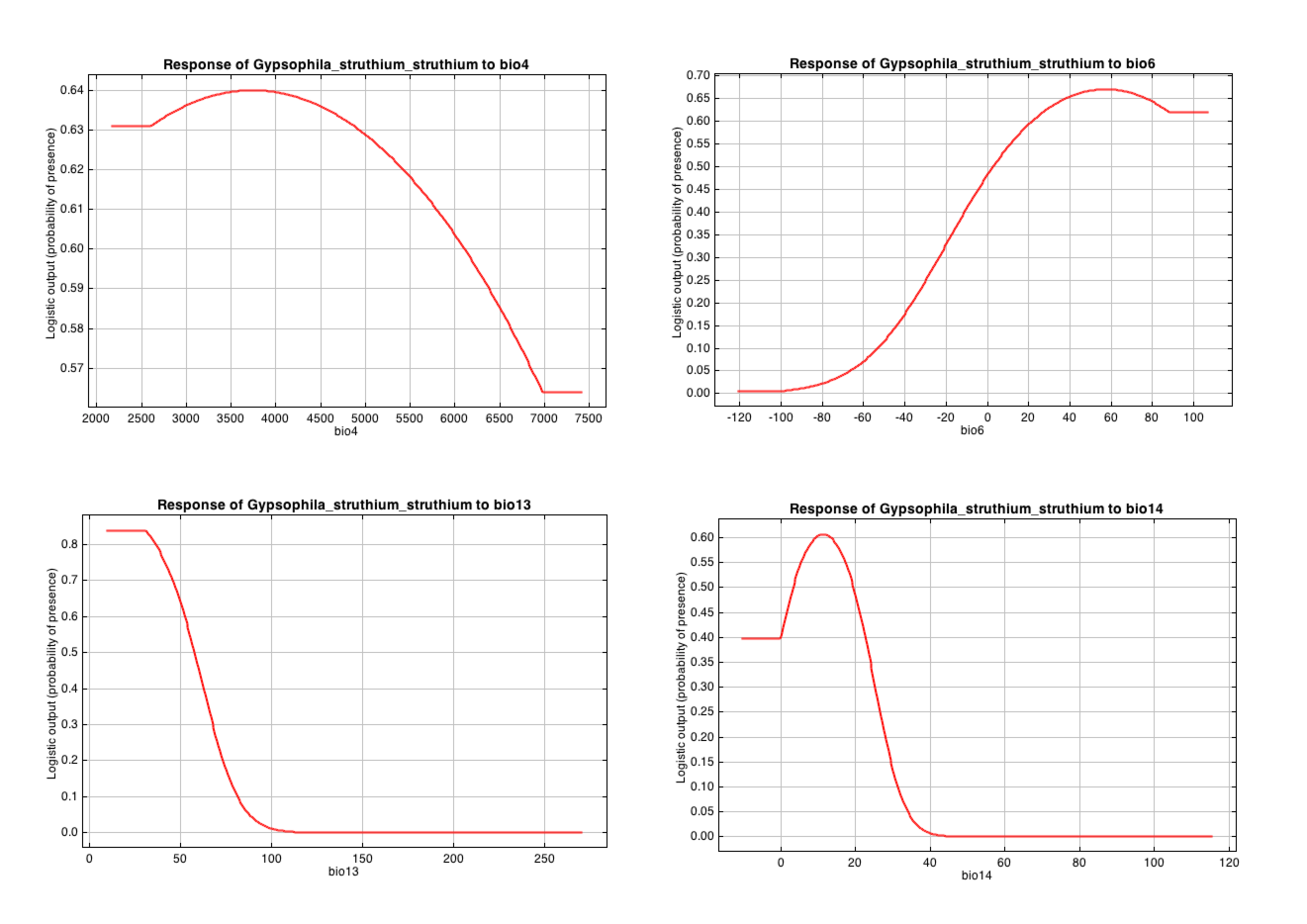

Supplement: S2 Fig — (TIF) [file pone.0190536.s002.tif]

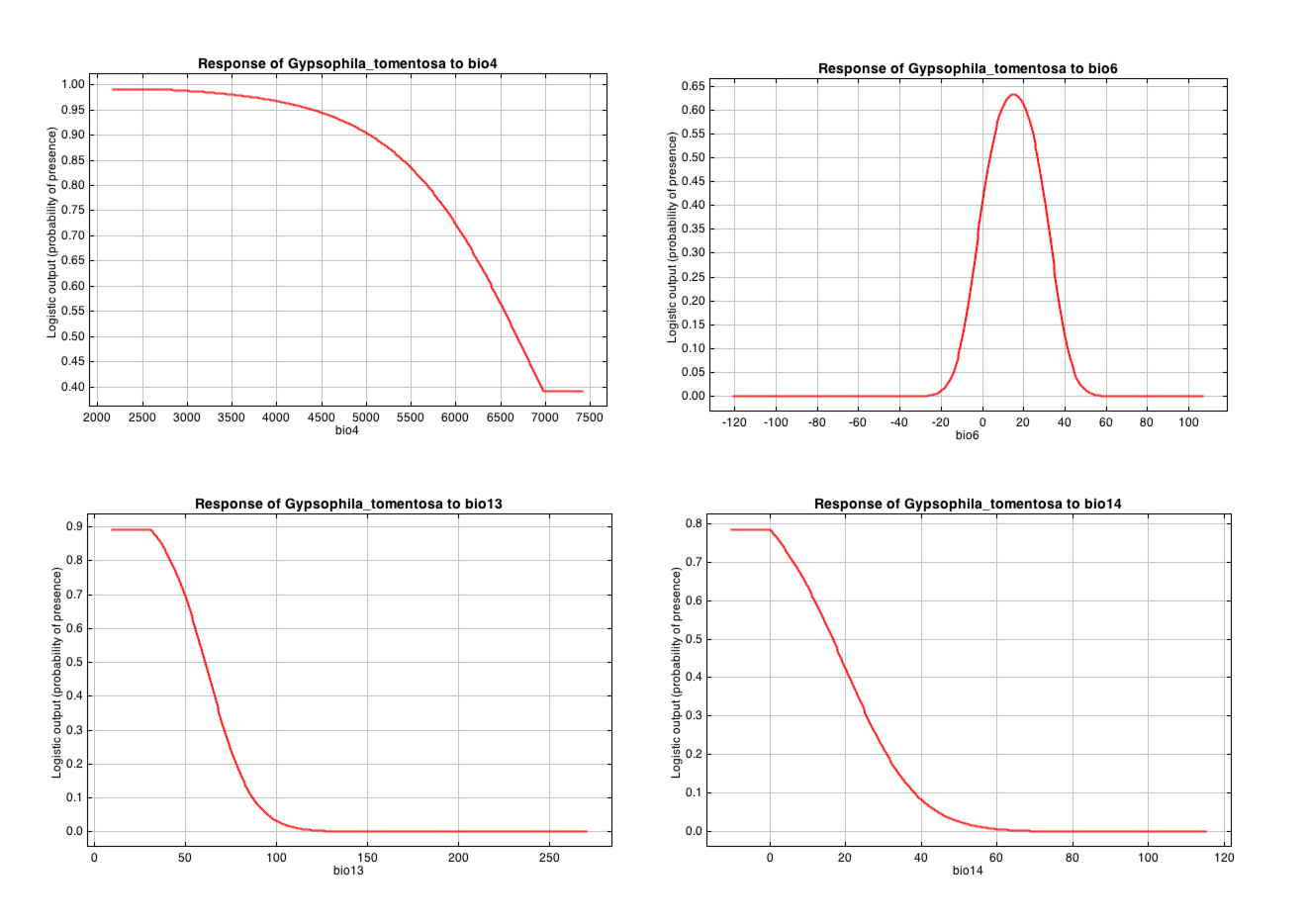

Supplement: S3 Fig — (TIF) [file pone.0190536.s003.tif]
